# Results from a 2018 cross-sectional survey in Tokyo, Osaka and Sendai to assess tobacco and nicotine product usage after the introduction of heated tobacco products (HTPs) in Japan

**DOI:** 10.1186/s12954-020-00374-3

**Published:** 2020-05-26

**Authors:** Jason Adamson, Claudia Kanitscheider, Krishna Prasad, Oscar M. Camacho, Elisabeth Beyerlein, Yoga Keralapura Bhagavan, Christopher Proctor, James Murphy

**Affiliations:** 1 Research & Development, British American Tobacco, Southampton, SO15 8TL UK; 2 Kantar Germany, Landsberger Straße 284, 80687 Munich, DE Germany

**Keywords:** Post-market surveillance, Cross-sectional survey, Heated tobacco products (HTPs), Tobacco harm reduction

## Abstract

**Background:**

For novel tobacco products that potentially reduce the risk of tobacco harm, post-market surveillance is important to observe population usage and behaviours associated with everyday use. This pilot study was performed to examine the use of tobacco products in three Japanese urban regions.

**Methods:**

This study was a cross-sectional epidemiological survey administered in Sendai, Tokyo and Osaka, Japan, from May 19th to June 25th, 2018. Participants were selected with a three-stage probability random sampling process that first identified primary sampling units, then households and finally individuals. Eligible participants were aged at least 20 years who were willing to participate after information about the study was provided. People younger than 20 years and those living in institutions were excluded. Questionnaires were paper based and administered door to door.

**Results:**

Responses were obtained from 4154 participants. Sixty-five percent self-reported being never, 19% current and 16% former users of any tobacco product at the time of the survey. Combustible tobacco products (almost all being cigarette) were used most (16%) followed by HTPs (5%). In the categories of combustible tobacco users and HTP users, 70% and 16%, respectively, used these products exclusively. Dual use was reported by 11% of respondents. Compared with 12 months before the survey, 12% of sole combustible tobacco products users were using HTPs exclusively or as dual users and 6% had quit tobacco products completely; 94% of sole HTP users remained sole users and 4% had quit tobacco products completely; and amongst dual users 12% had reverted to exclusive use of combustible tobacco products, 14% had switched to sole use of HTPs and 4% had quit tobacco products completely.

**Conclusion:**

HTPs seem to be accepted as an alternative tobacco product amongst combustible tobacco users. Given complex findings for dual use, improved understanding of the motivations underlying this behaviour would be of interest.

## Background

For adult smokers who wish to reduce cigarette consumption or for those who have tried to quit smoking but failed, while nicotine-replacement therapy is widely available, non-combustible tobacco and nicotine products such as heated tobacco products (HTPs), electronic cigarettes (e-cigarettes) and oral tobacco products, might offer other opportunities for tobacco harm reduction [4, 13, 21]. HTPs were introduced to the market in 1988, but were not initially successful [25]. HTPs contain tobacco that is mechanically heated without combustion, or via the passing of hot air. Thus, the aerosol released does not contain products of combustion [5, 25, 26]. The maximum heating temperature of 350 ^o^C results in reduced toxicant emissions [8, 12, 24] and less exposure of users to harmful or potentially harmful constituents [9, 24] compared with cigarettes smoke, which reaches 950 ^o^C within the lit end.

Japan is one of the world’s major markets for HTPs, with several product options available [5, 18, 26, 29]. However, usage patterns by consumers and the fundamental risks of these products need to be clearly contextualised within everyday use to evaluate the public health effects.

For tobacco harm reduction to be successful, products should demonstrate reduced harm to individual tobacco smokers and at the population level. Ideally, this would be achieved by a complete switch from cigarette smoking to products with potentially reduced harm. When a new product is launched in a market, it is important to understand how consumer behaviours change over time. Post-market surveillance studies allow observation of the product use at different timepoints and improve understanding of product acceptance [1].

This pilot study was performed to examine the current use of combustible tobacco products and HTPs in three cities in Japan and to examine changes in usage over 12 months.

## Methods

### Study design

This was an epidemiological population survey. The study protocol [1] and surveillance tool (10.17605/OSF.IO/JECDN) were approved by an independent ethics committee in Japan prior to commencing data collection.

### Selection of participants

This study was performed in three areas of Japan: Tokyo, Osaka and Sendai. These areas were selected to capture high numbers of current HTP users; Tokyo and Osaka are two largest metropolitan areas in Japan and Sendai was the launch city of an HTP.

Participants were selected with a three-stage probability sampling process. First, primary sampling units (PSUs) were selected by random sampling of street blocks from the Basic Resident Registration population data published by the Japanese Ministry of Internal Affairs and Communications, stratified by study city. Five hundred PSUs were initially selected, with the number in each area being proportionate to the population density. These PSUs were listed in ascending order of municipality codes and chosen for inclusion with randomly selected starting numbers that were no greater than the skip interval (calculated as the target population divided by number of PSUs in each stratum).

In the second stage, all households were listed by street number, in ascending order, using the Zenrin residential map database (Zenrin Co Ltd, Kitakyushu, Fukuoka, Japan). The first household was selected randomly, and 50 households per PSU with regular numeric intervals were chosen for interviewers to attend.

The next birthday method [23] was used as the third stage to select one individual per household to participate. People were eligible to participate if they were aged at least 20 years (the legal age to consume tobacco in Japan), living in private households, able to speak and read Japanese, and willing to participate after information about the study was provided. Exclusion criteria included people younger than 20 years and people currently living in prisons, military bases, mental facilities, or homes for the elderly.

### Data collection and processing

Study teams visited homes with a paper-based questionnaire that participants were asked to self-complete (unless they had visual impairments or difficulty writing, in which case the interviewer assisted). Data were collected for use of tobacco products (current, former or never) on sociodemographic status, age, sex, education level, employment status, household income and marital status. Use of tobacco products was defined as having consumed least 100 cigarettes or equivalents for other tobacco or nicotine products (HTPs, e-cigarettes, oral tobacco products and nicotine-replacement therapies) during the respondent’s lifetime. Combustible tobacco products included manufactured or roll-your-own cigarettes, cigars and cigarillos and/or pipes.

For combustible tobacco products and HTPs, additional information was requested on duration and frequency of product use, amount consumed, flavour preferences, tar level (cigarettes), quit attempts and use at ’12 months before’ and at the time of the survey. We asked current and former tobacco users about awareness of the foremost brands of HTP available in the study areas at that time: iQOS (Phillip Morris International, Neuchatel, Switzerland), glo (British American Tobacco, London, UK) and Ploom TECH (Japan Tobacco Inc., Tokyo, Japan). It was not deemed appropriate to ask never users of combustible tobacco products about awareness of branded tobacco products.

Data from the paper questionnaires and answers to semi-open questions translated into English were entered into an electronic data capture system via double data entry [1]. No imputation was applied for missing data. Weighting was applied to adjust for selection probabilities and for non-response according to population characteristics (region, age and sex).

### Measures

Intention to quit cigarette smoking and HTP use amongst current users was measured with the contemplation ladder [2], in which 0 indicates no thought of quitting and 10 indicates taking action to quit. For all current and former regular HTP users, reasons for HTP use were assessed with tools identified in the literature ([16]; PMI MRTPA THS-PBA-07 Rescreening Questionnaire [22];).

Self-reported dependency on nicotine was estimated with the Heaviness of Smoking Index. The index measures number of cigarettes smoked per day (1–10, 0 points; 11–20, 1 point; 21–30, 2 points; or ≥ 31, 3 points) and the time to first cigarette after waking (≤5 minutes, 3 points; 6-30, 2 points; 31–60, 1 point; and ≥ 61 min, 0 points), which are considered important predictors of quitting smoking [3]. The overall score is a composite of the scores from the two questions, giving a classification of low (0–2), medium (3–4) or high (5–6) nicotine dependence.

### Analyses

A sample size of 4000 participants (*n* = 20 households per PSU) was considered sufficient for subgroup analysis. An additional 150 participants were included for the 20–24-year age group because concerns have been raised about susceptibility to tobacco product use amongst young people [6] and about HTPs being viewed as ‘high tech’ and therefore having youth appeal [10], and because previous fieldwork experiences indicate that young people are under-represented with the applied sampling method. Thus, the final sample size was set at 4150.

All analyses presented herein are descriptive in nature and were performed with SAS 9.4. Categorical variables were analysed by frequency tables and continuous variables were reported by summary statistics. Data are presented for the total study population and stratified by age (in 5-year age groups from 20 to 79 years, and one group for people aged ≥ 80 years), sex and tobacco user status (never, former or current user). Differences between 12 months before and the time of the survey are also stratified by product type. In this report, weighted results are presented.

## Results

### Sociodemographics of study participants

Surveys were completed by 4154 participants from May 19th to June 25th 2018 (Tokyo *n* = 3261, Osaka *n* = 789 and Sendai *n* = 104; of which 4001 surveys were across all age groups, with a booster sample of 153 participants in the 20–24-year age group). Overall population characteristics are presented in Table 1.

**Table 1:** Population characteristics of study participants by tobacco use status (*N* = 4154)

Parameter	Never user total	Current user total	Former user total
Total	2697.9 (64.9%)	779.4 (18.8%)	674.5 (16.2%)
Gender			
Male	947.2 (35.1%)	565.7 (72.6%)	526.5 (78.1%)
Female	1750.6 (64.9%)	213.7 (27.4%)	148.1 (21.9%)
Age (years)			
Mean (SE)	51.4 (0.55)	48.0 (0.64)	57.8 (0.65)
Median	49.2	46.1	58.8
Min	20	20	20
Max	98	87	88
Highest level of education–*n* (%)			
Junior high school	180.4 (6.7%)	69.4 (8.9%)	44.1 (6.5%)
High school	901.1 (33.4%)	346.7 (44.5%)	254.6 (37.7%)
Professional training college	360.1 (13.3%)	105.0 (13.5%)	83.1 (12.3%)
Junior college	316.7 (11.7%)	27.1 (3.5%)	23.0 (3.4%)
College, university or graduate course	858.5 (31.8%)	210.8 (27.0%)	258.4 (38.3%)
Prefer not to answer	78.5 (2.9%)	20.3 (2.6%)	11.3 (1.7%)
Missing	2.5 (0.1%)	0.0 (0.0%)	0.0 (0.0%)
Employment status–*n* (%)			
Agriculture, forestry or fisheries	5.7 (0.2%)	0.9 (0.1%)	1.4 (0.2%)
Self-employed, family business, professional	255.2 (9.5%)	121.1 (15.5%)	118.3 (17.5%)
Regular employee	727.9 (27.0%)	362.9 (46.6%)	232.2 (34.4%)
Non-regular employee (part-timer)	520.3 (19.3%)	127.5 (16.4%)	87.2 (12.9%)
Unemployed	174.1 (6.5%)	31.3 (4.0%)	41.2 (6.1%)
Student	96.6 (3.6%)	8.7 (1.1%)	1.7 (0.3%)
Full-time homemaker	591.7 (21.9%)	52.6 (6.8%)	49.6 (7.4%)
Pensioner	259.4 (9.6%)	53.3 (6.8%)	131.8 (19.5%)
Prefer not to answer	65.3 (2.4%)	21.0 (2.7%)	11.1 (1.7%)
Missing	1.7 (0.1%)	0.0 (0.0%)	0.0 (0.0%)
Marital status–*n* (%)			
Never married	597.5 (22.1%)	164.1 (21.0%)	59.4 (8.8%)
Married	1838.1 (68.1%)	529.9 (68.0%)	553.2 (82.0%)
Cohabiting	4.5 (0.2%)	6.1 (0.8%)	1.0 (0.1%)
Widowed	173.7 (6.4%)	20.8 (2.7%)	25.6 (3.8%)
Divorced	47.0 (1.7%)	43.7 (5.6%)	25.5 (3.8%)
Prefer not to answer	37.1 (1.4%)	14.8 (1.9%)	9.8 (1.5%)
Missing	0.0 (0.0%)	0.0 (0.0%)	0.0 (0.0%)
Annual household income–*n* (%)			
< 3 million yen	406.0 (15.1%)	112.4 (14.4%)	111.4 (16.5%)
3 million to < 5 million yen	533.5 (19.8%)	202.4 (26.0%)	194.0 (28.8%)
5 million to < 10 million yen	736.7 (27.3%)	250.5 (32.1%)	196.7 (29.2%)
≥ 10 million yen	192.3 (7.1%)	63.9 (8.2%)	58.7 (8.7%)
No income	38.9 (1.4%)	4.1 (0.5%)	3.9 (0.6%)
I do not know	319.5 (11.8%)	45.3 (5.8%)	18.0 (2.7%)
Prefer not to answer	469.2 (17.4%)	100.8 (12.9%)	90.6 (13.4%)
Missing	1.7 (0.1%)	0.0 (0.0%)	1.3 (0.2%)

Prevalence is weighted to account for additional participants in the 20–24-year age group

### Tobacco and nicotine product usage

Sixty-five percent of respondents identified themselves as never, 19% as current and 16% as former users of any tobacco products at the time of the survey. The prevalence of never use was higher for females (83%) than for males (46%). The most frequently used products were roll-your-own cigarettes (16%), followed by HTPs (5%), with low use of e-cigarettes, cigars or cigarillos and pipes (latter four product types all < 0.5%).

### Manufactured and roll your own cigarette usage

Data were available on cigarette use for 642 participants. Most (92%) reported daily use and smoked on average 16 cigarettes per day. The average daily cigarette consumption for females (13) was slightly lower than for males (16).

Amongst daily cigarette smokers, 29% reported that they first smoked within 5 min of waking and 39% within 6–30 min. The percentage of participants who smoked within 5 min after waking up was similar in females (32%) and males (28%). Nicotine dependency scores were available for 589 current daily cigarette smokers, and were high for 6%, medium for 53% and low for 41%. Fewer females had high dependency than males (2% versus 8%).

Current cigarette smokers reported that they preferred no added flavour (48%), followed by menthol (36%) and other flavours (16%). Females had a higher preference for menthol cigarettes than males (47% versus 31%). The most common cigarette tar level was 1–3 mg (38%), followed by 4–6 mg (24%), 7–9 mg (19%) and 10 mg or higher (17%). More females preferred cigarettes with tar levels of 1-6 mg than males (75% versus 58%).

Amongst current combustible tobacco product users, half had scores that indicated no thought of quitting or considering quitting someday. More than half of respondents had tried to quit and 10% were taking action to quit (Table 2).

**Table 2:** Intention to quit smoking and other quitting characteristics for current cigarette and roll your own users in 2018

Parameter	*n* (%)
Intention to quit scale	642.0 (100.0%)
0 = no thought of quitting	159.3 (24.8%)
1	32.3 (5.0%)
2 = think I need to consider quitting someday	168.6 (26.3%)
3	12.7 (2.0%)
4	13.4 (2.1%)
5 = think I should quit but am not quite ready	116.4 (18.1%)
6	11.5 (1.8%)
7	9.9 (1.5%)
8 = starting to think about how to change my smoking patterns	41.5 (6.5%)
9	4.8 (0.7%)
10 = taking action to quit (i.e. cutting down)	64.8 (10.1%)
Missing	7.0 (1.1%)
Ever tried to quit	
No	298.4 (46.5%)
Yes	339.6 (52.9%)
Missing	4.0 (0.6%)
When was the last quit attempt (of those who ever tried to quit)	339.6 (100.0%)
< 3 months ago	49.5 (14.6%)
3–6 months ago	22.2 (6.5%)
6–12 months ago	22.5 (6.6%)
> 12 months ago	240.1 (70.7%)
Missing	5.3 (1.6%)
How long was the last quit attempt	339.6 (100.0%)
< 1 day	48.0 (14.1%)
1–7 days	118.5 (34.9%)
> 7 days–< 30	46.4 (13.7%)
> 30 days–< 6 months	52.0 (15.3%)
> 6 months–< 1 year	25.1 (7.4%)
≥ 1 year	46.0 (13.5%)
Missing	3.6 (1.1%)

Prevalence is weighted to account for additional participants in the 20–24-year age group

### HTPs

Of the 779 participants who were current tobacco product users, 81% had heard of iQOS, 55% of glo and 45% of Ploom TECH. Half (51%) had tried HTPs at least once. Of the 254 respondents who identified themselves as HTP users, most were users of iQOS (67%), followed by glo (18%) and Ploom TECH (16%), and most used them daily (iQOS 85%; glo 72%; Ploom TECH 52%). The proportion of current daily iQOS users was similar for men and women (69% and 67%, respectively), for glo use it was higher for women (86%) than for men (66%) and for Ploom TECH use was higher for men (19%) than for women (9%).

Daily iQOS users consumed on average 15 sticks per day, glo users 13 sticks per day and Ploom TECH users 3 tobacco capsules per day (equivalent to 12–18 conventional cigarettes) [15]. Amongst all current HTP users, the preferred flavour was menthol (62%), followed by regular (35%) and other flavours (3%). Females showed a slightly higher preference for menthol than males (68% versus 60%).

When asked about reasons of HTP use, most HTP users selected ‘reduced harm to people around them and themselves compared with conventional cigarettes’. Only around 10% indicated use to cut back smoking cigarettes or to quit overall smoking (Table 3).

**Table 3:** Reasons for HTP use amongst HTP users (multiple responses)

Reasons	Total–*n* (%)	Male–*n* (%)	Female–*n* (%)
Total	253.6 (100.0%)	184.8 (100.0%)	68.8 (100.0%)
They might be less harmful to people around me than conventional cigarettes	168.5 (66.4%)	121.8 (65.9%)	46.7 (67.9%)
They might be less harmful to me than conventional cigarettes	154.7 (61.0%)	116.3 (62.9%)	38.4 (55.8%)
They produce no ash	137.1 (54.1%)	98.8 (53.4%)	38.4 (55.8%)
HTPs do not smell bad	93.5 (36.9%)	67.6 (36.6%)	25.9 (37.7%)
HTPs contain no tar	81.7 (32.2%)	61.4 (33.2%)	20.3 (29.5%)
I was curious about HTPs	75.0 (29.6%)	56.3 (30.5%)	18.7 (27.1%)
HTPs do not bother people who do not use tobacco	74.6 (29.4%)	63.4 (34.3%)	11.2 (16.3%)
It helps me to cope with stress and to relax	48.8 (19.2%)	33.1 (17.9%)	15.7 (22.9%)
I have a friend or family member who uses HTPs	47.5 (18.7%)	27.7 (15.0%)	19.8 (28.8%)
I can use them in places where smoking conventional cigarettes is not allowed	42.6 (16.8%)	29.3 (15.8%)	13.3 (19.4%)
Using a HTP feels like smoking a conventional cigarette	41.4 (16.3%)	28.1 (15.2%)	13.3 (19.4%)
HTPs can help me cut back on smoking conventional cigarettes	32.1 (12.7%)	25.3 (13.7%)	6.8 (9.9%)
HTPs can help me quit smoking	22.8 (9.0%)	13.0 (7.0%)	9.9 (14.3%)
HTPs are new and innovative products	17.7 (7.0%)	13.8 (7.5%)	3.9 (5.7%)
They deliver a real tobacco taste	16.4 (6.5%)	8.6 (4.6%)	7.8 (11.4%)
They help me deal with cravings to smoke	16.3 (6.4%)	14.5 (7.9%)	1.7 (2.5%)
Out of habit	16.1 (6.4%)	11.1 (6.0%)	5.1 (7.4%)
Other reason*	5.2 (2.0%)	2.1 (1.1%)	3.1 (4.5%)

Prevalences are weighted to account for the additional participants in the 20–24-year age group *Free text option such as ‘someone gave it to me’ or ‘they are cleaner’

### Patterns of usage

Amongst current tobacco users at the time of the survey, sole use of manufactured or roll-your-own cigarettes was reported by 70% and sole use of HTP by 16%. Rates in men and women were similar for sole use of manufactured or roll-your-own cigarettes (68% and 71%, respectively), but more women than men used HTPs (21% versus 15%). Sole use of HTPs was highest in the age groups 25–29 years (23%) and 30–39 years (28%). Dual use of manufactured or roll-your-own cigarettes and HTPs was reported by 11% of participants at the time of the survey and was more common in males (12%) than females (8%).

Usage data for tobacco products at the time of the survey and 12 months previously were available for 791 participants (Table 4). Twelve percent of current combustible tobacco product users 12 months ago reported having started to use HTPs within the previous 12 months. The initiation of HTP use was higher for females than males and in the three age groups between 25 and 49 years, than in the 20–24-year age groups and ≥ 50-year age groups. The initiation rate per HTP was greatest for iQOS (8%), followed by glo (3%) and Ploom TECH (3%), with some participants using multiple HTPs.

**Table 4:** Change in use behaviour 12 months ago and current (2018) (*N* = 791)

Tobacco usage behaviour 12 months ago	Tobacco usage behaviour today–% (May/June 2018)				
	Total *n* (100 %)	Current CTP only user *n* (% per row)	Current HTP only user *n* (% per row)	Current dual user *n* (% per row)	Former tobacco user *n* (% per row)
	791.3 (100.0%)	533.6 (67.4%)	115.6 (14.6%)	83.5 (10.5%)	42.2 (5.3%)
Used only combustible tobacco products 12 months ago					
All Male Female	643.9 (100.0%) 470.2 (100.0%) 173.6 (100.0%)	522.6 (81.2%) 384.1 (81.7%) 138.5 (79.8%)	33.4 (5.2%) 20.6 (4.4%) 12.8 (7.4%)	42.5 (6.6%) 32.5 (6.9%) 10.0 (5.8%)	36.5 (5.7%) 27.5 (5.8%) 9.0 (5.2%)
Used only HTPs 12 months ago					
All Male Female	77.4 (100.0%) 54.9 (100.0%) 22.5 (100.0%)	0.0 (0.0%) 0.0 (0.0%) 0.0 (0.0%)	72.6 (93.8%) 51.0 (93.0%) 21.6 (95.8%)	0.0 (0.0%) 0.0 (0.0%) 0.0 (0.0%)	3.2 (4.1%) 2.2 (4.1%) 0.9 (4.2%)
Dual user 12 months ago					
All Male Female	60.9 (100.0%) 48.3 (100.0%) 12.6 (100.0%)	7.3 (11.9%) 7.3 (15.0%) 0.0 (0.0%)	8.4 (13.7%) 3.4 (7.0%) 5.0 (39.6%)	41.0 (67.3%) 34.7 (71.8%) 6.3 (50.1%)	2.5 (4.1%) 1.2 (2.5%) 1.3 (10.4%)

Prevalence is weighted to account for additional participants in the 20–24-year age group. Some consumers transitioned to use behaviours not shown in the table

Amongst sole users of combustible tobacco products 12 months before the survey, 5% had switched to exclusive HTP use (Table 4). A complete switch was observed from more women than men (7% versus 4%). The highest switching rate was observed in the age group 25–29 years (8%). Seven percent of sole combustible tobacco users 12 months before the survey had switched to dual use with HTPs. Switching to dual use was slightly more frequent amongst males (7%) than in females (6%) and was most common in the 25–49-year age groups. Within the previous 12 months, 5% of sole combustible tobacco product users had quit their use of tobacco products completely (Table 4). Most (94%) participants using only HTPs 12 months before the survey continued to do so. Four percent had quit tobacco use completely.

Over two-thirds of dual users 12 months before the survey had maintained this behaviour at the time of the survey (67%), 14% switched to using only HTPs and 4% quit tobacco use completely (Table 4). However, 12% of dual users switched back to solely using combustible tobacco products. Stratified by sex, 72% of male dual users were still dual users after 12 months, 7% switched to HTPs alone and 15% switched back to using only combustible tobacco products. For female dual users, the percentages were 50%, 40% and 0%, respectively.

Amongst never users of combustible tobacco products 12 months before the survey, 0.1% had started using HTPs and 0.2% had started using combustible tobacco products by the time of the survey. For former tobacco users 12 months before the survey, 1% re-initiated the use of a tobacco product, but all with HTPs. This rate was higher in females (4%) than in males (0.6%).

Amongst HTP users 12 months ago, 10 users reported having never used any combustible tobacco products. Amongst these, none had switched to sole or dual use of any combustible tobacco product at the time of the survey. In addition, no participants who were former sole combustible tobacco product users but had switched completely to HTPs 12 months before the interview reported reverting to cigarette smoking, alone or as dual use, at the time of the interview.

## Discussion

Japan is a country characterised by a high tobacco use prevalence which reached a peak in the mid-1970s with a cigarette smoking prevalence of ~ 75% amongst men; this rate has been decreasing constantly over the years, while women showed a rather stable smoking prevalence of 10–15% within this time period [7]. Prevalence of tobacco use in Japan is still high, with 18% of men being current smokers as of 2017 [27]. Until HTPs were introduced in Japan in 2014, other tobacco and nicotine product use frequency was low. Reports now suggest that Japan has the most developed HTP market of all countries worldwide, accounting for 85% of HTP global share [30, 31]. Evaluation of usage patterns after the introduction of novel tobacco/nicotine products is central to assess harm reduction at a population level. This study assessed use of HTPs and combustible tobacco products in three regions in Japan and investigated differences in use 12 months apart as well as differences in use by sex and age. At the time of the survey, around two-thirds of tobacco users exclusively used combustible tobacco products (almost all were cigarettes, except for three participants who used cigar/cigarillo/pipe); this was followed by exclusive use of HTPs then dual use of both product types.

Uptake of HTPs was greatest amongst women and users aged 25–49 years. Similar patterns of use for novel tobacco technologies have been found elsewhere, amongst younger and more educated groups [20]. The main reason given in this study for use of HTPs on a regular basis was potential reductions to harm and annoyance to others, followed by harm reduction to self (the current user). This finding is consistent with other studies, where it was noted that Japan has strong cultural values of order, cleanliness, quality and respect for others [10]. Only a small proportion of respondents indicated that they used HTPs to cut back on or quit cigarette smoking—similar to the proportion who had a clear intention to quit smoking (both 10%).

### Changes in use behaviour amongst tobacco users

Compared with 12 months before the survey, while 81% of people who used combustible tobacco products continued to do so at the time of the survey, around 12% had initiated using HTPs regularly, whether alone or as dual users. Most participants who had completely switched to HTPs 12 months before the survey continued to use only HTPs. Of note, no participants who had used only HTPs 12 months ago, regardless if they were never or former combustible tobacco users, switched back to combustible tobacco use either alone or as dual use, potentially indicating a stable behaviour.

For dual users, a more complex situation was observed. Although most who were dual users 12 months before the survey maintained dual use, 14% had switched completely to HTPs, of which more were women than men. Men were more likely than women to stop dual use and revert to using only combustible tobacco products (15% versus 0%). Further research is needed to identify the reasons for differences in behaviour in dual users.

The substantial decline of cigarette consumption could be attributable to the initiated implementation of tobacco control actions as well as to an increased awareness of health risks and diseases caused by combustible tobacco use. There is also some evidence suggesting the decline and replacement effect on cigarette sales due to HTP introduction in Japan. A study from Stoklosa et al. [28] showed that cigarette sales were relatively stable before the introduction of HTPs in Japan (2% average annual decline from 2011 to 2015) but fell significantly afterwards (10% average annual decline from 2015 to 2018). They noted an immediate regional effect in each prefecture HTPs were launched and did not find any alternative explanations for the change.

### Initiation of HTPs from tobacco non-users

It is important to consider whether new tobacco products with a perceived reduced risk of harm are appealing to people who have never used tobacco or nicotine products or were a former user. In this study, 13 participants showed HTP use without a history of using combustible tobacco products. Around 1% of respondents who were former tobacco users 12 months before interviews began to use a tobacco product again, but all used HTPs. These findings suggest that the introduction of an alternative tobacco product will not generally lead to uptake amongst tobacco non-users. Amongst never and former tobacco users aged 20–24 years, none started using HTPs in the 12 months before the study, and the overall HTP usage in the previous 12 months was lower within this age group than in the 25–49-year age groups.

### Pilot study limitations and strengths

This study has several limitations. It considered only three urban regions in Japan in order to test the approach before nationwide assessment, and therefore results herein may not be generalisable to the rest of the country or other countries. Whether differences in cultural values, product acceptance, the regulatory environment and the availability of a larger variety of novel tobacco products affect other markets requires more assessment. As with any self-reported survey, recall bias is a potential issue and that should be considered whilst interpreting the data. Lastly, the questionnaire used in this pilot study was not designed to target the motivations of dual users compared with sole users of HTPs. Potential differences in motivations and behaviours would be an area of interest for future studies of current HTP users.

The study design and resulting data present various strengths. First, it was able to estimate product-specific prevalence and collect data on tobacco and nicotine product consumption. Second, the stratified three-stage sampling method yielded representative populations in the three selected study areas. Third, as data collection was by self-administered questionnaire, this minimised bias that could arise from social desirability for answers.

For external validation, results were compared with tobacco prevalence data from Japan Tobacco’s Annual Survey 2018 [11], data published by the Japanese Ministry of Health, Labour and Welfare [14] and Japanese smoking prevalence data published by the Foundation for a Smoke-Free World [19]. Results herein were in line with those data and, therefore, appear to be representative data for the study regions.

## Conclusion

We found that 27% of tobacco consumers used HTPs, either alone or in parallel with combustible tobacco products. Most HTP users indicated that they felt HTPs might be less harmful to people around them or to themselves compared to combustible cigarettes. Only small percentages saw HTPs as a route to quitting or cutting back consumption of combustible tobacco use. No respondents who had been using HTPs alone 12 months before the survey were using combustible tobacco products at the time of the survey, and 4% had quit tobacco completely. Thus, HTPs seem to be accepted as an alternative tobacco product amongst combustible tobacco users. Uptake by never users of tobacco products was minimal for both combustible tobacco products and HTPs. These findings supported the initiation of a further study into nationwide data. Improved understanding of the motivations underlying dual use of HTPs and combustible tobacco products would be of interest.

## Data Availability

The study protocol and instrument have been published (protocol [1]; instrument questionnaire http://www.doi.org search.10.17605/OSF.IO/JECDN).

## Abbreviations

AE: Adverse event/effect
BAT: British American Tobacco
CTP: Combustible tobacco product
FMC: Factory made cigarette
HSI: Heaviness of smoking index
HTP: Heated tobacco product
IEC: Independent Ethics Committee
KH: Kantar (Health Division)
RYO: Roll your own (cigarette)
